# On the Various Risks of Operations

**Published:** 1868-03

**Authors:** James Paget


### SELECTED ARTICLES.

---

### ARTICLE I.

### *On the Various Risks of Operations.*

By James Paget, F.R.S., (delivered at St. Bartholomew's Hospital.)

(Concluded.)

There are many patients to whom you cannot assign a morbid constitution, but who are feeble in all their processes. No organ, it may be, works wrongly; but no organ works with due power. Many children are in this condition, and some adults, whose condition has been admirably portrayed by Dr. Chambers in his book on Italy. They are not always bad subjects for operation. Repair will probably take place in them as feebly as any other vital process; but I believe they are not particularly liable to the disease after operations from which the greatest risks arise. Children of this class you should be cautious of operating upon for harelip or other such defects as do not urgently require interference, and in adults, if you can defer operations to some period of better health, you should do so; but all this for fear of local failure rather than of incurring any unusual risk of life. For in the management of these, as of all cases, you will find that the chief vital risks of operations are not through mere defects of power, but through disease. The measure of danger is not in the proportion between more or less of vital force, and more or less of exhaustion, but in the amount of liability to real disease of the blood and tissues.

You often hear me speak of patients as "cold-blooded." I do not know that the whole of their blood is less warm than that of ordinary persons, but some of it is, for their hands and feet are seldom or never naturally warm; and some of them feel, when you touch them, as cold as reptiles in the same climate—their hands and feet feel as moist and damp as toads and frogs. The circulation in all these cold parts is of course very slow, and probably it has not a due velocity in any of their textures; for wherever you can see vascular parts in them, they are of duller tint than they should be, dusky, and with a purple hue rather than a rosy one; and with these signs you find small pulses, and general indications of slowness in all vital processes. They digest slowly, and are very prone to constipation; and the women amongst them menstruate disorderly, and are liable to headaches and backaches, and a variety of nervous symptoms. People of this kind are so numerous that you will do well to look out for them among your cases, and to treat them specially with iron, with particular regard to this cold-bloodedness and slowness of life. They are not bad subjects for operation; rather, I should reckon them amongst the good ones; for they have always seemed to me singularly little liable to fall into the troubles of erysipelas, or pyæmia, or any other disorders of the blood: and the healing of their wounds is apt to be interrupted. Observe their defects; minister to them with warmth and good food, but not high stimulants or great eating, and they will do as well as any you will have.

And, to finish this account of the influence of diseased, or disorderly constitutions on the results of operations, let me tell you of the people that are commonly called "nervous." I do not refer to those with manifest disease in any part of their nervous system, but to those that are exceedingly sensitive, mobile, and excitable, whether in their sensitive or motor organs—who are very emotional, and with their whole cerebro-spinal nervous system altogether too alert. You will find them and their friends always apprehensive of the results of operations; they will tell you that they are so nervous they can bear no shock; and they look with the greatest apprehension upon the inflictions of any injury. All this is fallacious. You may be surprised at observing how very little influence upon their organic processes this excessive vivacity of their cerebro-spinal system exercises. Time after time I have found patients who have complained of agonies in their wounds, and I do not doubt have felt them, but whose pulses have been unmoved. They have had enormous pain, but no fever, no single sign of disturbance of their general nutrition; they have had spasmodic movements of their limbs, tremblings, and rigors, but no mischief has followed. Besides, the same mobility of mind which makes these patients very fearful before an operation makes them hopeful directly after it; and amongst all the people that can in any sense be called invalids, I know none who more generally pass through the consequences of operation with impunity than do those who are commonly called nervous, and whose nervousness consists, if I may use the expression, in too great a vivacity of their whole cerebro-spinal system.

Sometimes you may be forced to operate during the continuance of an acute disease; and although the circumstances of the case may give you little choice as to whether you shall operate or not, it is well to be aware of the degree in which the acute disease may influence the result of your proceedings.

Patients with ague bear operations as well as others of the same class; but, in the course of their recovery, they may alarm you by having one or more ague-fits, exactly resembling those that precede pyæmia. And more than this: if a patient has ever had ague, and even many years afterwards, you perform an operation on him, ague may seem to be renewed in him at some short time after the shock, or loss of blood, or whatever other damage he may have sustained. I have so often noticed this, that whenever I hear of severe rigors following on any operation, I ask for a previous history of ague ; and I have sometimes found that the patient has almost forgotten it in the long lapse of time since he suffered from it.

The question of amputation often arises when the patient is suffering with erysipelas, or with that spreading inflammation of the cellular tissue which is closely akin to erysipelas. I have often said to you that upon a secondary amputation as a confession of either a mistake or a disappointment. Either a primary amputation ought to have been done, and by mistake it was left undone ; or if for any apparently sufficient reason it was not done, the necessity of doing, the secondary amputation implies the disappointment of just hopes. I have spoken with this disparagement of secondary amputations because the necessity for them is so likely to come when the probability of success is reduced by the operation being performed while the patient is in acute disease. I cannot tell you the numerical increase of risk ; but I believe that the mortality after amputations during erysipelas, or spreading cellular inflammation, would be found very much greater than that of primary amputations, or of secondary amputations done for merely wasting suppuration or irreparable local damage. I scarcely know any set of cases in which I have operated with less hope than in those of compound fracture, or similar injuries, in which the question is raised whether a patient, who seems dying with acute disease, may have what is called a chance of his life by amputation. In the large majority of such cases the chance by operation seems to be less than that of keeping the patient alive by the ordinary treatment of erysipelas, or whatever other acute disease he may be suffering with.

What are the chances of recovery from operations done during pyæmia. I think I can answer safely, that in acute pyæmia in which the patient has rigors once or more in a few days, and profuse sweatings, with very rapid pulse and breathing, and with delirium, and rapid wasting, or with dry tongue and yellowness of skin, or any considerable number of these symptoms, the probability of good is so small, and of harm so great, that you should refuse to operate. But in chronic pyæmia, when the disease requiring operation adds largely to the exhaustion from which the patient is suffering, the removal of the disease may be very proper. Suppose, for example, a patient with a crushed foot or a crushed hand, in whom signs of acute pyæmia have recently appeared. Whatever be the state of the injured part, I would not add the damage of an amputation to the burden that the patient already has to bear. But if the pyæmia have become chronic, attended with only wasting and sweating, and the formation of abscesses here and there, and if the injured part be manifestly useless, and a source of irritation or of exhaustion, the mere existence of pyæmia in the chronic form would not turn me from the operation required by the part. The occasions for operating in any other than these acute diseases are not many, but in diptheria or croup you may have to perform tracheotomy, and during peritonitis a hernia may require operation. These are all cases of necessity, and their results are not materially affected by the general acuteness of the disease. If their local good is accomplished, the healing of the wound and the recovery of the patient may occur as any case, unless indeed, (which I have never seen), a wound after tracheotomy should itself become diphtheritic.—(*Lancet.*)

